# Effectiveness of early start of direct hemoperfusion with polymyxin B-immobilized fiber columns judging from stabilization in circulatory dynamics in surgical treatment patients

**DOI:** 10.4103/0972-5229.63032

**Published:** 2010

**Authors:** Yuichiro Sakamoto, Kunihiro Mashiko, Toru Obata, Hisashi Matsumoto, Yoshiaki Hara, Noriyoshi Kutsukata, Hiroyuki Yokota

**Affiliations:** **From:** Department of Emergency and Critical Care Medicine, Chiba-Hokusoh Hospital, Nippon Medical School, Japan; 1 **From:** Department of Molecular Cell Biology, Institute of DNA Medicine, Jikei University School of Medicine, Japan; 2 **From:** Department of Emergency and Critical Care Medicine, Nippon Medical School, Japan

**Keywords:** Early goal directed therapy, polymyxin B-immobilized fiber column (PMX), septic shock

## Abstract

**Background:**

Septic shock remains a major cause of multiple organ failure and is associated with a high mortality rate. In 1994, direct hemoperfusion using a polymyxin B-immobilized fiber column (PMX; Toray Industries Inc., Tokyo Japan) was developed in Japan and has since been used for the treatment of septic shock arising from endotoxemia.

**Materials and Method:**

We treated 36 patients with septic shock using direct hemoperfusion with PMX. The patients were analyzed in two groups based on whether they had undergone surgery prior to DHP-PMX treatment (surgical group: surgical treatment before DHP-PMX, medical group: no surgical treatment). In surgical group, DHP-PMX was started within three hours after the surgical treatment. Various factors were measured before and after DHP-PMX.

**Results:**

The mean Acute Physiology and Chronic Health Evaluation (APACHE) II score was 27.4 ± 8.8, and the mean sepsis-related organ failure assessment (SOFA) score was 11.8 ± 4.9 before DHP-PMX. The SOFA score was significantly higher (*P* = 0.0091) and the PaO2/FiO2 ratio (P/F ratio) was significantly lower (*P* = 0.0037) in medical group than in surgical group prior to DHP-PMX. A chi-square test showed that the survival rate in surgical group was significantly better than in medical group (*P* = 0.0027). The survival rate of surgical group (84.2%) was judged to be very good because the predicated survival rate based on the APACHE II score (25.0) was only 46.5%. On the other hand, the survival rate of medical group (35.3%) was almost equal to that predicted by the APACHE II score (30.6; predicted survival rate, 27.4%).

**Conclusion:**

The results of this study suggest the utility of early DHP-PMX in surgical group.

## Introduction

Severe sepsis is made a diagnosis when sepsis is responsible for some organ dysfunction. Despite advances in intensive care medicine, the mortality rate of patients with severe sepsis remains at over 30%.[1]

In 1994, however, direct hemoperfusion using a polymyxin B immobilized fiber column (DHP-PMX; Toray Industries Inc., Tokyo Japan) was developed in Japan and has since been used for the control of endotoxemia in patients with septic shock. The use of a polymixin B immobilized fiber column has been shown to reduce serum endotoxin levels.[2] But, adsorption materials using a DHP-PMX are not proved completely. Several clinical reports describing the effectiveness of this column have also been reported; for example, promising clinical data showing an increase in the systolic blood pressure (SBP) and an improved PaO2/FiO2 ratio.[3] The diminishment of inflammatory cytokine levels and other mediators after DHP-PMX has also been reported.[4–7] As for the DHP-PMX, clinical trials are now being performed in many countries including Italy, Spain, Russia and. India.

A recent systematic review of a pooled sample of 1,390 patients showed that DHP-PMX therapy significantly lowered endotoxin levels, improved blood pressure, and reduced mortality.[8] There are still few reports about start time of DHP-PMX and the characteristic of the effective cases. Here, we confirmed the characteristic of the effective case and reviewed a utility for the early DHP-PMX start within postoperative (surgical group) or post diagnosis (medical group) three hours.

## Materials and Methods

We treated 36 patients with septic shock using direct hemoperfusion with PMX. Hemoperfusion was performed over a period of two hours for each column, and DHP-PMX was performed twice. The patients were analyzed in two groups based on whether they had undergone surgery prior to DHP-PMX treatment (surgical group: surgical treatment before DHP-PMX was started within three hours of the, medical group: no surgical treatment before DHP-PMX). Primary disease of surgical group was indication of surgical treatment such as the peritonitis, and medical group was not surgical cases such as the pneumonia. We weighed an effect of DHP-PMX compared with Acute Physiology and Chronic Health Evaluation (APACHE) II predicted death rate in each group, and considered effectiveness of DHP-PMX for the adjuvant therapy of sepsis in early diagnostic phase each. We divided septic shock cases into two groups than primary disease, but it is not the examination that compared two groups. We compared with outcome of each groups and APACHE II predicted death rate because we reviewed the appropriateness of the procedure of the DHP-PMX. This DHP-PMX procedure was two columns continuous enforcement in hope for maximum effect at early phase.

High mobility group box-1 (HMGB-1), N-arachidonoylethanolamine (AEA), 2-arachidonoyl glycerol (2-AG), plasminogen activator inhibitor-1 (PAI-1) and F2-isoprostane were measured before and immediately after DHP-PMX, and at first and third days after DHP-PMX. Blood samples were collected using a heparinized syringe, and were immediately de-proteinized by mixing with a 10-times excess volume of acetonitrile. After the addition of internal standards (deuterated AEA, 2-AG, and F2-isoprostane, 10 ng each), the acetonitrile solution was filtered to remove blood protein debris. The organic solvent was then removed using a centrifugal concentrator *in vacuo* at 40°C. The resulting precipitate was stored at −80°C until LC/MS preparation.

The blood extract precipitates were resolved using 1-mL of methanol and were added to an equal volume of chloroform and 0.875 volume of acidic water (0.01 N HCl in aqueous solution). After vigorous mixing for 30 seconds, the heavy organic layer remaining after centrifugation was collected and dried using a centrifugal concentrator in vacuo at 40° C. The precipitation was redisolved with methanol for tandem mass assay.

Endocannabinoids and F2-isoprostane were detected using a liquid chromatography tandem mass spectrometry (LC/MS/MS) system (Q-trap; Applied Biosystems, Foster City, CA, USA) with the isotope dilution method. The HMGB-1 level was measured using an enzyme-linked immunosorbent assay (ELISA) (Shino-Test Corporation, Japan). Interleukin-6 (IL-6) and plasminogen activator inhibitor-1 (PAI-1) levels were measured using enzyme immunoassays (EIAs). These parameters were measured before and immediately after DHP-PMX. The APACHE II score[9] and the sepsis-related organ failure assessment (SOFA) score[10] were also evaluated at the of admission to the intensive care unit.

Blood access for DHP-PMX was established using a double-lumen catheter inserted into the femoral vein using the Seldinger method. Hemoperfusion was performed for two hours at a flow rate of 80 mL/min, and DHP-PMX was performed twice continuously. Nafamostat mesilate (Torii Co., Ltd, Tokyo, Japan) was used for anticoagulation.

The approval of our institution's ethical committee and informed consent were obtained. The results were expressed as the mean ± SD. Differences were analyzed using the Wilcoxon generalized test or, the chi-squared test, and the Kaplan-Meier survival curves were compared using a logrank test. A *P*-value less than 0.05 were regarded as statistically significant. Predicted survival rate was calculated by APACHE II predicted death rate estimated methods.

## Results

**Figure 1:**
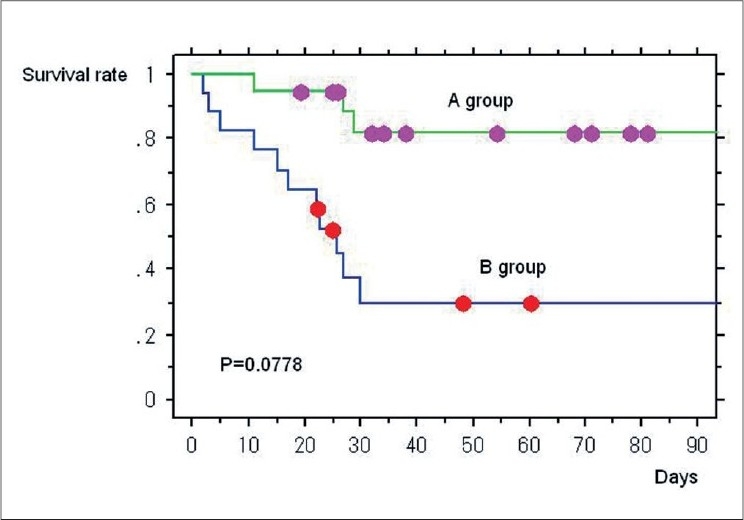
The Kaplan Mayer survival curves indicated a better outcome in surgical treatment before DHP-PMX group (A) than in no surgical treatment group (B), although the difference was not significant (*P* = 0.0778).

**Figure 2:**
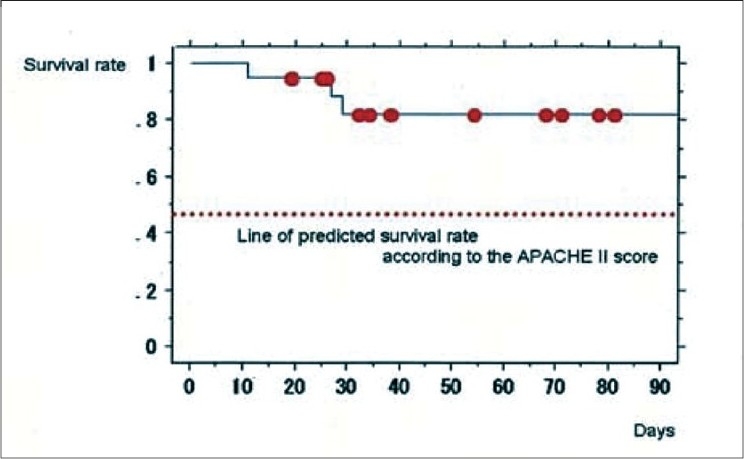
The survival rate of surgical treatment before DHP-PMX group (A) (84.2%) was judged to be very good because the predicted survival rate based on the APACHE II score (25.0) was only 46.5%.

**Figure 3:**
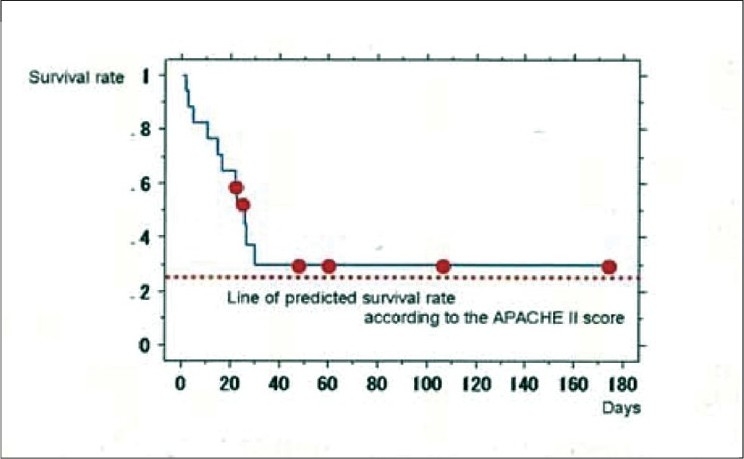
The survival rate of non surgical treatment group (B) (35.3%) was almost equal to that predicted by the APACHE II score (30.6; predicted survival rate, 27.4%).

Surgical group contained 19 patients and medical group contained 17, respectively. The average patient age was 61.0 ± 13.5 years; 22 of the patients were men and 14 were women. The mean APACHE II score was 27.4 ± 8.8, and the mean SOFA score was 11.8 ± 4.9 before DHP-PMX. Twenty-two patients survived and 14 patients died. We showed primary disease two groups in Table 1. The backgrounds and severity scores of surgical group and medical group are shown in Table 2. The SOFA score was significantly higher (*P* = 0.0091) and the PaO2/FiO2 ratio (P/F ratio) was significantly lower (*P* = 0.0037) in medical group than in surgical group prior to DHP-PMX. A chi-square test showed that the survival rate in surgical group was significantly better than in medical group (*P* = 0.0027). The Kaplan Mayer survival curves indicated a better outcome in surgical group than in medical group, although the difference was not significant (*P* = 0.0778) [Figure 1]. The survival rate of surgical group (84.2%) was judged to be very good because the predicted survival rate based on the APACHE II score (25.0) was only 46.5% [Figure 2]. On the other hand, the survival rate of medical group (35.3%) was almost equal to that predicted by the APACHE II score (30.6; predicted survival rate, 27.4%) [Figure 3]. The changes of several factors and the clinical conditions, before and after DHP-PMX are shown in Table 3. The changes of IL-6, PAI-1, AEA, 2-AG, HMGB-1, and F2-isoprostane were not significantly different between surgical group and medical group [Table 3]. Only the systolic blood pressure level was significantly higher in surgical group than in medical group (*P* = 0.0203). The rate of improvement to a blood pressure of over 30 mmHg was 78.9% in surgical group. A significant decrease in the platelet level after DHP-PMX treatment was noted in all cases in surgical group [Table 4].

**Table 1:** Primary disease

Disease	Group-A	Group-B
Peritonitis	14	0
Pleuritis	4	0
Gas gangrene	1	1
Pneumonia	0	12
Meningitis	0	1
Hemolitic streptococcal infection	0	2
Purulent arthritis	0	1

**Table 2:** Background and disease severity surgical group or medical group

Characteristics	Surgical group	Medical group	*P* value
No. of patients	19	17	NS
Sex (male/female)	12/7	10/7	NS
Age (years), mean+SE	61 ± 15	61 ± 12	NS
APACHE II score (mean +SE)	25.0 ± 7.5	30.6 ± 9.5	NS
SOFA score (mean +SE)	9.8 ± 4.1	14.2 ± 4.8	0.0091
PaO2/Fio2 (mean +SE)	326 ± 1.34	18.4 ± 1.15	0.0037
Survival/expired (suvival rate)	16/3 (84.2%)	6/11 (35.3%)	0.0027

**Table 3:** Improved rate of any factors and condition before and after PMX-DHP

After DHP-PMX	Surgical group	Medical group	*P* value
Decrease of IL-6	53.2% (12/19)	70.6%(12/17)	NS
Decrease of PAI-1	57.9% (11/19)	64.7%(11/17)	NS
Decrease of AEA	58.4% (13/19)	64.7%(11/17)	NS
Decrease of 2-AG	57.9% (11/19)	82.3%(14/17)	NS
Decrease of HMGB-1	63.2% (12/19)	41.2%(7/17)	NS
Decrease of F2Isoplostan	52.6% (10/19)	35.3%(6/17)	NS
Decrease of SOFA score	42.1% (8/19)	11.8%(2/17)	NS
Increase of Pa02/Fio2	36.8% (7/19)	41.2%(7/17)	NS
Increase of SBP (over 30 mmhg)	78.9%	41.2%(717)	0.0203

**Table 4:** Change of platelet count by PMX-DHP therapy

	Before PMX-DHP	After PMX-DHP	*P* value NS
A group	15.8±8.8	11.9±6.8	0.0091
B group	9.6±7.4	8.0±8.4	0.0652
All cases	12.7±8.6	10.1±7.7	0.0013

## Discussion

A multicenter prospective randomized controlled pilot trial of DHP-PMX has suggested that DHP-PMX treatment may improve cardiac and renal dysfunction caused by sepsis or septic shock.[11] In a systematic review study, DHP-PMX appeared to reduce endotoxin levels effectively and to have possible effects on improvement of blood pressure and gas exchange, and on reduction of vasoactive agents usage and mortality.[3] Several factors are removed by DHP-PMX treatment, particularly endocannabinoids (AEA and 2-AG), HMGB-1 and IL-6. We previously showed a relation between hemodynamic improvement and HMGB-1 in serum levels, and between improvement of respiratory functions and 2-AG and PAI-1 levels in septic shock patients who underwent DHP-PMX.[7]

In 2008, the international SSCG committee published new international guidelines for the management of severe sepsis and septic shock.[12] These new guidelines recommend the standard protocol for the resuscitation of cases with sepsis-induced shock. This resuscitation is directed toward achieving the previously mentioned goal for the initial six-hour period after the diagnosis of septic shock. One of the goals of early goal-directed resuscitation is a mean arterial pressure of over 65 mmHg. We feel that this recommendation shows the importance of early treatment for improving circulatory dynamics, such as arterial pressure. In the present study, patients who had undergone surgical treatment prior to DHP-PMX had very good outcomes (compared with the predicted outcomes based on the APACHE II scores) and improved systolic blood pressures. The improvement in blood pressure in the present study was greater than that for a review study, possibly reason in differences in the treatment method and/or the timing of treatment. In the present study, hemoperfusion was performed over a period of two hours for each column, and DHP-PMX was performed twice; additionally, DHP-PMX was started within three hours of surgical treatment. Because the patients who underwent surgery had very good outcomes after DHP-PMX, we suspect that the early induction of DHP-PMX is a very important point; this rationale is supported by EGDT. In the surgical group, the source of infection is removed; in non-surgical cases, however, gaining control of the source of infection can take some time. Thus, this parameter likely has a large effect on the clinical condition. Many sepsis connection factors were not difference between two groups (surgical indication cases and non surgical cases), clinical condition were very difference and we should have changed the way of the adjuvant therapy such as the DHP-PMX each group. New treatment methods should be explored for non-surgical cases. Long time enforcement and twice continuously approach should be examined, along with other treatment factors, in the future.

Thus, a potential theoretical limitation of interpretation of our study are that the difference in mortality of both groups was statistically significant with chi-square test, but not with Kaplan-Mayer analysis. Our investigation group was small number and the numbers of patients gave the power to obtain valid results from this chi-square test, however each survival rate judging from APACHE II score in two groups might be supporting results even this study was retrospective. Other limitation point is the point that both groups had a substantially different profile of underlying disease conditions for telling about potential effects of polymyxin-B hemoperfusion. At the same time, to clear the characteristic clinical condition of treatment resistive cases is very important to improve the outcome of the septic shock cases.

This report describes the utility of early DHP-PMX introduction as a component of stabilization in circulatory dynamics on surgical cases. Moreover, a further improvement in the survival rate might be possible if patients are consecutively treated using twice PMX column. However, patients with septic shock in whom surgery is not indicated, such as those with severe pneumonia, respond poorly to this therapy. Our previous paper showed a relation between the effectiveness of DHP-PMX therapy and the plasma levels of F2-isoprostane, an oxidative stress marker, in patients with severe pneumonia.[13] Further improvements in treatment strategies for such cases are needed.

Recently, early use of polymyxin B hemoperfusion in abdominal septic shock (the EUPHAS) randomized controlled trial was published, this paper showed that DHP-PMX therapy added to conventional therapy significantly improved hemodynamics and suggested a reduction in 28-day mortality in septic shock cases with intra-abdominal infection.[14] These results support our paper from the point that the outcome of the intra-abdominal infection disease that is a surgical cases, is good.

## Conclusion

Septic shock treatment using twice consecutive PMX columns is a useful component of early goal-directed therapy for surgical cases.

## References

[1] Angus D, Wax RS (2001). Epidemiology of sepsis: An update. Crit Care Med.

[2] Sato T, Shoji H, Koga N (2003). Endotoxin adsorption by polymyxin B immobilized fiber column in patients with systemic inflammatory response syndrome: The Japan experience. Ther Apher Dial.

[3] Cruz DN, Perazella MA, Bellomo R, de Cal M, Polanco N, Corradi V (2007). Effectiveness of polymyxin B-immobilized fiber column in sepsis: A systematic review. Crit Care.

[4] Tani T, Hanasawa K, Kodama M, Imaizumi H, Yonekawa M, Saito M (2001). Correlation between plasma endotoxin, plasma cytokines, and plasminogen activator inhibitor-1 in septic patients. World J Surg.

[5] Sakamoto Y, Mashiko K, Matsumoto H, Hara Y, Kutsukata N, Yamamoto Y (2007). Relationship between effect of polymyxin B-immobilized fiber and high mobility group box-1 protein in septic shock patients. ASAIO J.

[6] Sakamoto Y, Mashiko K, Matsumoto H, Hara Y, Kutsukata N, Takei K (2006). Effect of direct hemoperfusion with a polymyxin B immobilized fiber column on high mobility group box-1 (HMGB-1) in severe septic shock: Report of a Case. ASAIO J.

[7] Sakamoto Y, Mashiko K, Obata T, Matsumoto H, Hara Y, Kutsukata N (2007). Clinical responses and improvement of some laboratory parameters following polymyxin B-immobilized fiber treatment in septic shock. ASAIO J.

[8] Cruz DN, Bellomo R, Ronco C (2007). Clinical effects of polymyxin B-immobilized fiber column in septic patients. Contrib Nephrol.

[9] Knaus WA, Zimmerman JE, Wagner DP, Draper EA, Lawrence DE (1981). APACHE: Acute physiology and chronic health evaluation: A physiological based classification system. Crit Care Med.

[10] Vincent JL, Moreno R, Takala J, Willatts S, De Mendonca A, Bruining H (1996). The SOFA (Sepsis-related Organ Failure Assessment) score to describe organ dysfunction/failure: On behalf of the Working Group on Sepsis-Related Problems of the European Society of Intensive Care Medicine. Int Care Med.

[11] Vincent JL, Laterre PF, Cohen J, Burchardi H, Bruining H, Lerma FA (2005). A pilot-controlled study of a polymyxin b-immobilized hemoperfusion cartridge in patients with severe sepsis secondary to intra-abdominal infection. Shock.

[12] Dellinger RP, Levy MM, Carlet JM, Bion J, Parker MM, Jaeschke R (2008). Surviving Sepsis Campaign: International guidelines for management of severe sepsis and septic shock: 2008. Crit Care Med.

[13] Sakamoto Y, Mashiko K, Obata T, Matsumoto H, Hara Y, Kutsukata N (2008). Relationship between treatment resistance cases using polymyxin B-immobilized fiber and oxidative stress. ASAIO J.

[14] Cruz DN, Antonelli M, Fumagalli, Foltran F, Brienza N, Donati A (2009). Early use of polymyxin B hemoperfusion in abdominal septic shock: the EUPHAS randomized controlled trial. JAMA.

